# Follicle‐stimulating hormone worsens osteoarthritis by causing inflammation and chondrocyte dedifferentiation

**DOI:** 10.1002/2211-5463.13238

**Published:** 2021-07-12

**Authors:** Zhikun Huan, Yan Wang, Mengqi Zhang, Xiujuan Zhang, Yaping Liu, Lei Kong, Jin Xu

**Affiliations:** ^1^ Department of Endocrinology Shandong Provincial Hospital Cheeloo College of Medicine Shandong University Jinan China; ^2^ Shandong Provincial Key Laboratory of Endocrinology and Lipid Metabolism Jinan China; ^3^ Institute of Endocrinology and Metabolism Shandong Academy of Clinical Medicine Jinan China; ^4^ Department of Endocrinology Shandong Provincial Hospital Affiliated to Shandong First Medical University Jinan China; ^5^ Department of Endocrinology Jining No.1 People’s Hospital Jining China

**Keywords:** chondrocytes, dedifferentiation, follicle‐stimulating hormone, IL‐6, osteoarthritis, type I collagen

## Abstract

Previous studies have found follicle‐stimulating hormone (FSH) receptors on chondrocytes (cartilage cells), but the mechanism of FSH action on chondrocytes is not clear. The purpose of this experiment is to study whether FSH affects chondrocytes and how it causes changes in these cells. Our results show that osteoarthritis became worse after FSH injection in the knee joint of mice. After the stimulation of chondrocytes by FSH, a total of 664 up‐regulated genes, such as *Col12a1* and *Col1a1*, and 644 down‐regulated genes, such as *MGP*, were screened by transcriptomics. A subset of extracellular matrix (ECM)‐related genes and pathways underwent Gene Ontology (GO) and Kyoto Encyclopedia of Genes and Genomes (KEGG) enrichment analysis, and the downregulation of *MGP*, the upregulation of *EGR1* and *Col1a1*, and the increase of *IL‐6* were verified. It was also observed that FSH can inhibit the cAMP/PKA and MKK4/JNK signaling pathway. In conclusion, we demonstrated that FSH can increase cartilage inflammatory response and promote chondrocyte dedifferentiation by inhibiting the cAMP/PKA and MKK4/JNK signaling pathways.

## Graphical abstract

Here, we demonstrate through *in vivo* experiments, transcriptomic screening, and *in vitro* experiments that FSH can inhibit cAMP/PKA and MKK4/JNK signaling pathways and cause inflammatory response and dedifferentiation of chondrocytes, thereby aggravating osteoarthritis.

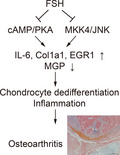

**Abbreviations**
ADAMTS: a disintegrin and metallo‐proteinase with thrombospondin motifs
DMM: destabilization of medial meniscus
ECM: extracellular matrix
FBS: fetal bovine serum
FSH: follicle‐stimulating hormone
FSHR: follicle‐stimulating hormone receptor
GO: gene ontology
KEGG: Kyoto Encyclopedia of Genes and Genomes
NS: normal saline
OA: osteoarthritis
PPI: protein–protein interaction
siRNA: small interfering RNA

Osteoarthritis (OA) is increasingly attracting attention as the most serious joint disease and one of the leading causes of disability among the elderly [1, 2]. It is reported that OA currently affects 250 million people worldwide, with a significantly higher prevalence among women when compared to men [3].

OA is a degenerative disease whose main symptom is joint pain, which is caused by various factors, such as fibrosis, cracking, ulceration, and loss of joint cartilage [4]. It can occur in various parts of the body, such as the lumbar spine, hip, and knee, with the knee being the most common site. However, the etiology of OA is currently unclear, and possible risk factors include age, female sex, obesity, inflammation, and so on [3, 4].

Chondrocytes are the only cellular component of adult articular cartilage and are therefore a central aspect of the study of the pathogenesis of OA [5]. Chondrocytes account for approximately 10% of the wet weight of articular cartilage, while the matrix secreted by articular cartilage accounts for 60%–85% of the wet weight of articular cartilage, and collagen and proteoglycans in the matrix account for 60% of the dry weight of cartilage [6]. In the physiological state, articular cartilage is maintained in a stable state by adjusting the balance between the extracellular matrix (ECM) components and their degradative enzymes [7]. In the pathological state, various degradative enzymes, such as a disintegrin and metallo‐proteinase with thrombospondin motifs (ADAMTS) or matrix metalloproteinases, cause an imbalance between chondrocyte anabolism and catabolism by degrading proteoglycans or collagen, leading to the development of OA [8, 9].

It is believed that decreased estrogen is responsible for the higher incidence of OA in postmenopausal women [10]. However, when estrogen levels are normal during the perimenopausal period, the incidence of OA is already higher in women compared to men, and follicle‐stimulating hormone (FSH) levels are often already elevated [11]. FSH, a hormone secreted by basophils in the anterior pituitary, is composed of glycoproteins. It can regulate the development of the human body, growth, puberty, sexual maturity, and a series of physiological processes related to reproduction. FSH plays a biological role through the follicle‐stimulating hormone receptor (FSHR). FSHR belongs to the G protein‐coupled receptor with seven transmembrane and is mainly expressed in the gonad. FSHR is also found to be expressed outside the gonad [12, 13]. Additionally, in previous studies, we found the expression of FSHR in chondrocytes [14], which provided a basis for the study of the effects of FSH on cartilage.

Therefore, we investigated the effect of FSH on OA by performing transcriptome sequencing and bioinformatics analysis on primary chondrocytes after FSH stimulation to check whether the transcript levels were changed and further to explore the mechanism of its occurrence.

## Materials and methods

### Animal model

A total of 24 7‐week‐old C57BL/6J female mice were purchased from Vital River (Beijing, China) and treated with 4 treatments: SHAM + NS (normal saline), SHAM + FSH, destabilization of medial meniscus (DMM) + NS, and DMM + FSH. Each treatment killed the mice after 4 and 8 weeks, respectively (*n* = 3). Each 3 mice were housed in one cage, and the environment was kept at constant temperature and humidity, with alternating day and night (12 h day and 12 h night), and adequate water and food supply was provided. DMM was performed by detaching the medial tibial collateral ligament of the right knee of the mouse and implementing the sham operation by only exposing the joint cavity, which was followed by suturing. The FSH used was recombinant human FSH (Goenafen, Germany, Merck) at an injectable dose of 20 IU·kg^−1^, approximately 5 µL per mouse in the knee joint cavity. The NS group was injected with 5 µL NS.

In accordance with the guidelines of the Shandong University Animal Protection and Use Committee, all animals were taken care of in a humane manner. This study was approved by the Experimental Animal Ethics Committee of the Shandong Provincial Hospital, Affiliated to Shandong University.

### Histological analysis and immunohistochemistry

Materials were taken from the right knee of mice, fixed with paraformaldehyde, tissue dehydrated, decalcified and paraffin embedded. Each cartilage sample was cut into thick sections of 5 µm. The sections were stained with safranin O‐fast green staining solution (Servicebio, Wuhan, China), images were acquired by using a Leica microscope with a LAS system, and the degree of OA was analyzed by using Mankin's score.

### Extraction and culture of primary chondrocytes

Fresh cartilage tissues were isolated from the knee joints of newborn mice and digested for 6 h at 37 °C in DMEM F12 medium (Hyclone, Logan, UT, USA), which contained 10% FBS (Gibco, New York, NY, USA), P/S (meilunbio, Dalian, China), cysteine and collagenase P (Sigma, St. Louis, MO, USA), and the digestion was terminated by adding the complete medium to the basal digest. The digest was then collected and centrifuged at 200 ***g*** for 15 min. The supernatant was removed, the precipitate was resuspended in 10 mL DMEM F12 + P/S + FBS, and the suspension was filtered through a 100 mm Falcon filter in a fresh 50 mL test tube. Next, the pellet was resuspended in DMEMF12 + P/S + FBS by centrifuging the tube at 200 ***g*** for 5 min, and the suspension was filtered through a 40 mm Falcon filter into the used 50 mL tube. The filter was flushed with 10 mL DMEMF12 + P/S + FBS, and the suspension was again filtered through the used filter. The centrifugation and suspension cycles were performed twice. Finally, chondrocytes were obtained and inoculated in 6‐well plates at 5 × 10^5^ cells·mL^−1^. Chondrocytes were cultured in a humidified environment supplemented with 10% FBS, 0.1% P/S, 50 mg·mL^−1^ l‐ascorbic acid (Gibco, New York, NY, USA), and cysteine solution (35.1 mg·mL^−1^) containing 5% carbon dioxide at 37 °C. The culture medium was changed every 2 days.

### Interference with FSHR

Primary chondrocytes were divided into four groups, that is Si NC (normal control) group, Si NC + FSH group, Si FSHR group and Si FSHR + FSH group, with three biological replicates in each group, according to whether FSHR was interfered with and the addition of FSH stimulation. When the cell density reached 80% or more, the cells were transfected with FSHR small interfering RNA (siRNA) or NC siRNA using Lipofectamine 3000 reagent (Invitrogen, Carlsbad, CA, USA). Transfection was then followed by the addition of FSH at a concentration of 25 ng·mL^−1^ to cells requiring FSH stimulation, and RNA was extracted half an hour after stimulation and sent to Novogene for transcriptome sequencing.

### Transcriptomics

#### Differential expression analysis

deseq2 software (1.16.1) (Bioconductor) was used for differential expression analysis between the two combinations of comparison. deseq2 provides a statistical program to determine the differential expression in digital gene expression data by using a model based on a negative binomial distribution. Therefore, Benjamini and Hochberg's method was used to adjust the resulting *P*‐value to control the rate of discovery of error. Genes with adjusted *P* < 0.05 were found to be differentially expressed by deseq2.

#### GO and KEGG enrichment analysis of differentially expressed genes

The GO enrichment analysis of differentially expressed genes was realized by the clusterprofiler r software (Bioconductor). The GO terms with corrected *P* < 0.05 were used as GO terms with significant enrichment of differentially expressed genes. KEGG is a database resource. We used the clusterprofiler software to analyze the statistical enrichment of differentially expressed genes in the KEGG pathway.

#### Interaction analysis of differential gene protein network

Protein–protein interaction (PPI) analysis of differentially expressed genes is based on STRING databases that know and predict PPIs. PPI analysis of differentially expressed genes is based on STRING databases that know and predict PPIs.

### Culture of ATDC5 cells

ATDC5 cells undifferentiated medium consisted of DMEM F12, 10% FBS (EVERY GREEN, Zhejiang, China), and P/S, and they were cultured at 37 °C with 5% CO_2_ in an incubator environment with fluid changes or passages every 2 days. To the cells before treatment, 10% ITS (Sigma) was added to the culture medium in order to induce cell differentiation into chondrocytes 14 days after the treatment of cells.

### FSH stimulates ATDC5 cells

FSH was purchased from the R&D system (Minneapolis, MN, USA) and diluted with sterile PBS. FSH stimulation concentrations were 0, 10, 25, and 50 ng·mL^−1^ for western blot validation of Col1a1, EGR1, and IL‐6 only. The rest of the experiments the concentration of FSH stimulation were 25 ng·mL^−1^.

### Protein extract and western blot

We needed to aspirate the medium, wash the cells 3 times with ice PBS, lyse the cells with the ratio of RIPA:PMSF: Phosphatase inhibitor cocktail A: Phosphatase inhibitor cocktail B being 98 : 1 : 0.5 : 0.5, put about 120 μL of lysis solution into 60 mm dishes, scrape off the cells and incubate on ice for 20 min, lyse the cells with ultrasonic and centrifuge at 4 000 ***g*** for 15 min, and after centrifugation, aspirate the supernatant and dispense the supernatant. Proteins were electrophoresed on 10% SDS gels and transferred to PVDF membranes, sealed with 5% skim milk, incubated with antibodies against Col1a1 (1 : 500 abcam, Cambridge, UK), EGR1 (1 : 1000 CST, Danvers, MA, USA), IL‐6 (1 : 2000 Proteintech, Wuhan, China), JNK (1 : 1000 CST), p‐JNK (1 : 1000 CST), GAPDH (1 : 5000 Proteintech, Wuhan, China), MKK4 (1 : 1000 abcam), and p‐MKK4 (1 : 5000 abcam) by using anti‐rabbit or anti‐mouse secondary antibodies in combination with primary antibodies. The membrane‐bound antibodies were detected using the Immobilon Western chemiluminescent HRP substrate (Millipore, Billerica, MA, USA) and analyzed by the imagej software (National Institutes of Health, Bethesda, MD, USA).

### RNA extraction and quantitative real‐time polymerase chain reaction analysis

The cultured cells from each experiment were washed three times with cold PBS and solubilized with TRIzol. The extracted RNA was measured for concentration, reverse transcribed, and subjected to quantitative real‐time PCR in a Roche LightCycler480 system by using the following cycle conditions: 95 °C for 5 min, 95 °C for 10 s, 60 °C for 10 s, and 72 °C for 10 s for 45 cycles. The entire reaction consisted of 1 μL cDNA (1000 ng), 1 μL primers, 10 μL Bestar Syb Green qPCR mastermix, and 8 μL ddH2O.

The primer sequences were as follows:

GAPDH: F:TGTCTCCTGCGACTTCAACA R:GGTGGTCCAGGGTTTCTTACT.

EGR1: F:TATGCTTGCCCTGTCGAGTC R:GGATGTGGGTGGTAAGGTGG.

Col1a1: F:TTCTCCTGGCAAAGACGGAC R:CTCAAGGTCACGGTCACGAA.

IL‐6: F:TCCTTCCTACCCCAATTTCCA R:GTCTTGGTCCTTAGCCACTCC.

MGP: F: CAAGCCTGCCTACGAGATCA R: TGCCTGAAGTAGCGGTTGTA.

#### Immunofluorescence

Cells were fixed in 4% paraformaldehyde and permeabilized with 0.5% Triton X‐100 for 10 min, followed by closure with 2% BSA for 1 h. Cells were incubated overnight at 4 °C using IL‐6 antibody (1 : 200 Proteintech, Wuhan, China). Secondary antibodies were incubated with goat anti‐mouse secondary antibody (1 : 1000 Invitrogen, Carlsbad, CA, USA) for 1 h at room temperature and protected from light. The final slices were sealed with a DAPI‐containing blocker (abcam) protected from light. Images were taken using a Laser Confocal Microscope (Leica, Wetzlar, Germany).

### cAMP ELISA assay

ATDC5 cells were treated with serum‐free medium containing 1% IBMX (Sigma) for 30 min at 80%–85% density and then FSH 25 ng·mL^−1^ was added or Forskolin stimulated for 1 h. The cells were then scraped off with 0.1 m HCl and centrifuged at 4 000 ***g*** for 10 min, after which the assay reagents were configured according to the ELISA kit (BioVision, Milpitas, CA, USA) instructions. Finally, the OD value at 450 nm was read and the camp concentration was calculated from it.

### Statistics analysis

Data from all of these experiments were analyzed by using graphpad prism 8 (GraphPad Software, San Diego, CA, USA) for unpaired Student's *t*‐tests. *P* < 0.05 indicates a statistically difference, and *P* < 0.01 indicates a statistically significant difference.

## Result

### FSH can worsen OA

**Fig. 1:**
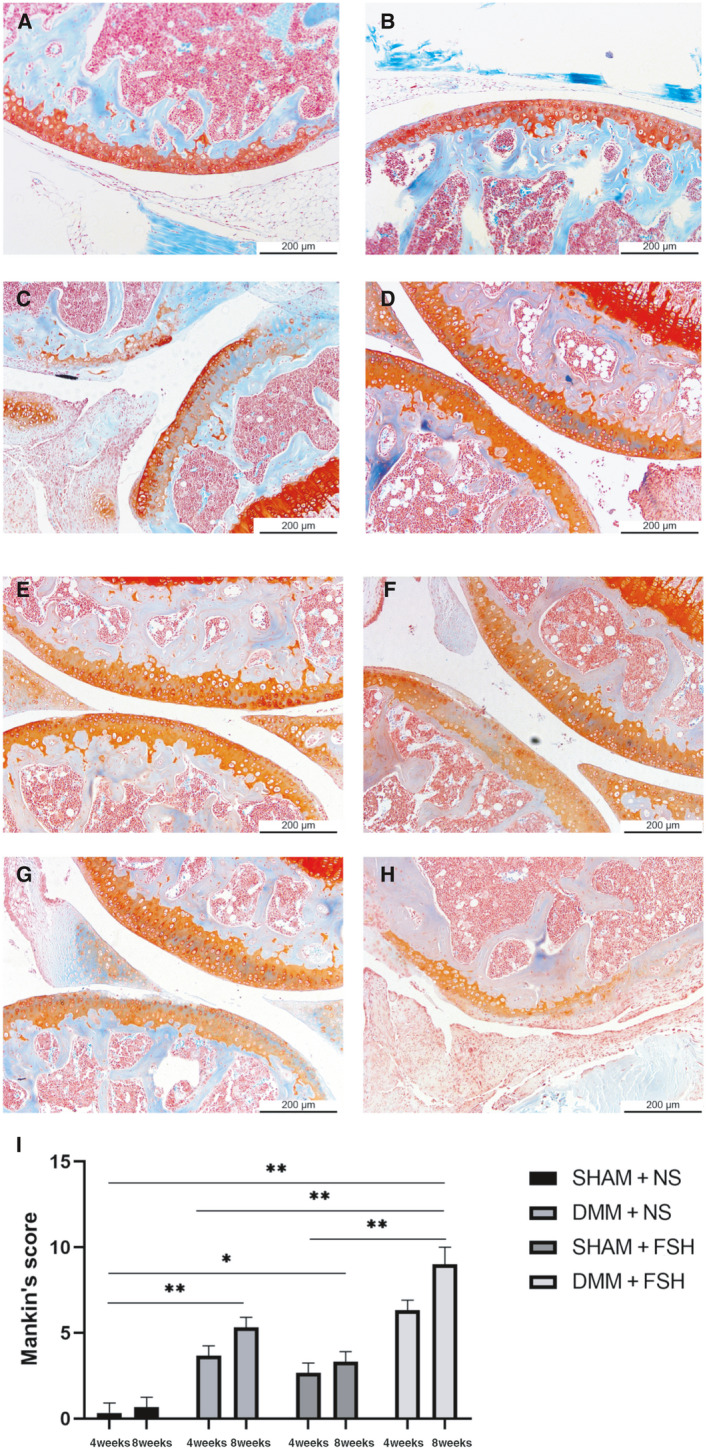
Animal modeling and OA scores. (A–D) Knee joint staining in the saline injection group. (A) SHAM + NS 4W, (B) SHAM + NS 8W, (C) DMM + NS 4W, (D) DMM + NS 8W. Scale bar: 200 μm. (E–H) Knee joint staining of the animals in the FSH injection group. (E) SHAM + FSH 4W, (F) SHAM + FSH 8W, (G) DMM + FSH 4W, (H) DMM + FSH 8W. Scale bar: 200 μm. (I) Comparison of Mankin's scores at 4W and 8W for various treatments. Statistical differences were determined by an unpaired Student’s *t*‐test (mean ± SEM, *n* = 3, *P < 0.05, **P < 0.01).

We first observed the effect of FSH on animals through surgical modeling and injection of FSH or NS and performed safranin O‐fast green staining on the knee sections. Our results revealed significant lesions in the knee joints of the mice after the DMM operation, and Mankin’s score was also significantly increased (refer to Fig. 1A–D,I). It proves that DMM surgery can successfully model OA. After the injection of FSH into the knee joint cavity of the mice, both the sham operation group and the operation group showed different degrees of joint damage, including the destruction of joint surface integrity and even the loss of normal joint structure, and the lesions were more severe than those in the saline injection group (refer to Fig. 1E–I). It was also observed that after the FSH injection, each group had higher Mankin’s scores than the saline injection group.

### Quantitative gene analysis and differential gene expression analysis

**Fig. 2:**
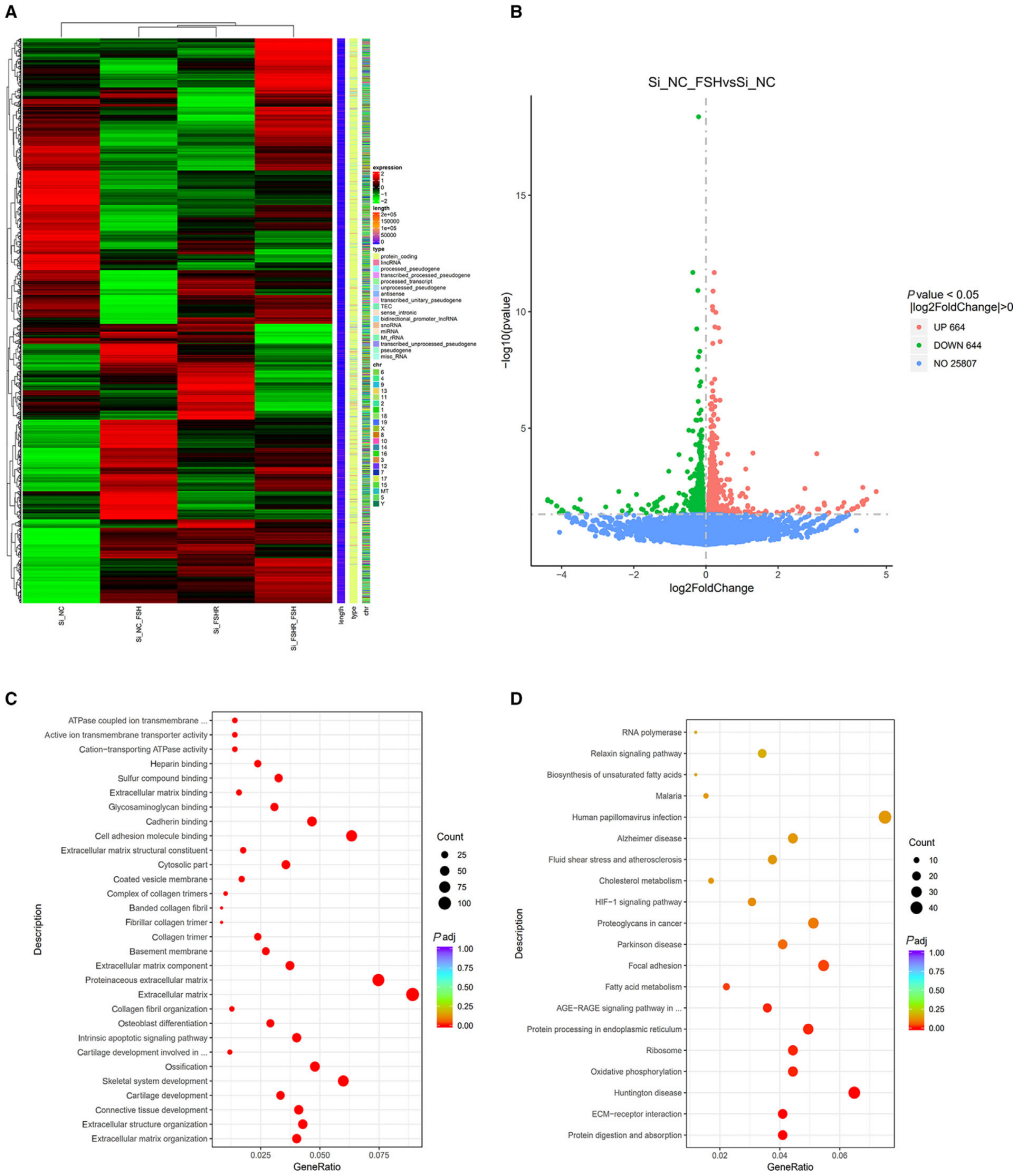
Differential gene expression analysis and GO/KEGG enrichment analysis. (A) The differential genes from all comparison groups are taken and then clustered together as a differential gene set. The differential gene set was subjected to cluster analysis, where genes with similar expression patterns were clustered together. (B) Volcano plots provide a representation of the differential gene distribution for each comparison combination, with horizontal coordinates indicating the genes in the treatment and control groups Fold change in expression (log2FoldChange). The vertical coordinates indicate differences in gene expression between the treatment and control groups of the level of significance (−log10padj or −log10pvalue). Up‐regulated genes are indicated by red dots and down‐regulated genes are indicated by green dots. (C) The horizontal coordinate is the ratio of the number of differential genes annotated to the Gene Ontology (GO) term to the total number of differential genes. The vertical coordinate is the GO term, the dot size represents the number of genes annotated to the GO term, and the colors from red to purple represent the significant size of enrichment. (D) The horizontal coordinate of the graph is the ratio of the number of differential genes annotated to the Kyoto Encyclopedia of Genes and Genomes (KEGG) pathway to the total number of differential genes, and the vertical coordinate is the KEGG pathway, with the size of the dots representing the number of genes.

Next, to investigate the mechanism of the effect of FSH on chondrocytes, we performed transcriptomic sequencing. The difference genes of all the comparison groups were taken as the difference gene set after merging (refer to Fig. 2A), and the cluster analysis of the difference gene set showed that the Si NC group was significantly different from the Si NC + FSH group. However, the difference between the Si FSHR group and the Si FSHR + FSH group was smaller after interfering with the FSHR. The differences between the Si NC group and the Si NC + FSH group were analyzed in focus (refer to Fig. 2B). There were a total of 1308 differential genes in the two groups, including 664 up‐regulated genes, such as *Col12a1, Col1a1, Col5a1, Col3a1, Egr1*, and 644 down‐regulated genes, such as *MGP, Hmgn2, Emb, Timp1, and Cd24a*. In order to further analyze the functions of the differential genes, we performed enrichment analysis of the differential genes to discover the biological pathways. A total of 6640 GO terms were enriched for differential genes in the Si NC group and the Si NC + FSH group (refer to Fig. 2C). The more significantly enriched GO term includes ECM organization, extracellular structure organization, cartilage development, etc. In addition, a total of 293 pathways were enriched for differential gene enrichment on KEGG (refer to Fig. 2D), and we observed that the more obvious KEGG pathways that were enriched included protein digestion and absorption, ECM‐receptor interaction, and so on.

### FSH increases the inflammatory response of chondrocytes and shows de‐differentiated behavior

**Fig. 3:**
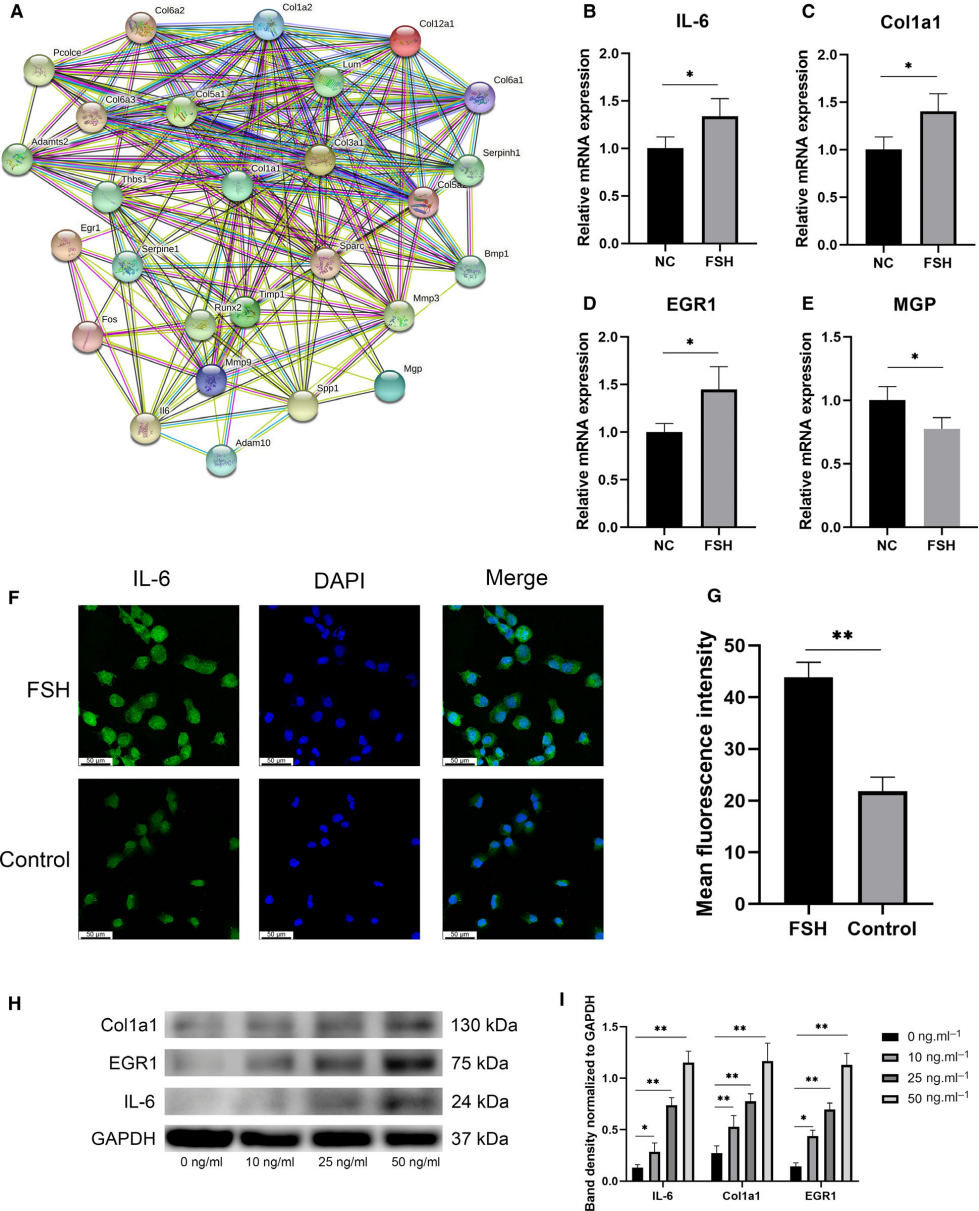
Validation of differential genes and IL‐6. (A) Network diagrams are drawn based on protein interactions, with proteins as nodes. (B–E) ATDC5 cells were stimulated with 25 ng·mL^−1^ FSH, and the expression of IL‐6, Col1a1, EGR1, and MGP was detected by qRT‐PCR, respectively (*n* = 4). (F) Immunofluorescence staining of IL‐6, magnification: 400x. Scale bar: 50 μm. (G) Comparison of mean fluorescence intensity between FSH group and control group. (H) The cells were stimulated with 0, 10, 25, and 50 ng·mL^−1^ FSH, and the expression of Col1a1, EGR1, and IL‐6 was observed by the western blot (*n* = 3). (I) The quantitative analysis of protein expression was performed by using imagej and comparison of differences. The statistical differences were determined by an unpaired Student’s *t*‐test (mean ± SEM, *P < 0.05, **P < 0.01).

In the protein interaction network analysis of differential genes, we found that the inflammatory factor *IL‐6* was closely associated with each collagen and other differential genes (refer to Fig. 3A). Therefore, we tried to verify whether IL‐6 changed after FSH stimulation of chondrocytes, and the results were consistent with our assumption that IL‐6 increased with higher FSH concentrations, both in terms of RNA levels and protein levels (refer to Fig. 3B,H,I). Immunofluorescence also showed higher fluorescence intensity in the FSH‐stimulated group, demonstrating that chondrocyte IL‐6 secretion did increase after FSH stimulation (refer to Fig. 3F,G). We also partially validated the transcriptomic results using the ATDC5 cell line and observed increased expression of EGR1, and the chondrocyte dedifferentiation marker Col1a1, and decreased MGP expression after FSH stimulation at the RNA level (refer to Fig. 3C–E). Similarly, a corresponding gradient change in EGR1, Col1a1 at different concentrations (0, 10, 25, 50 ng·mL^−1^) after the FSH stimulation of cells was observed at the protein level, which showed a dose–effect relationship (refer to Fig. 3H,I).

### FSH inhibits the cAMP/PKA pathway and the JNK pathway

**Fig. 4:**
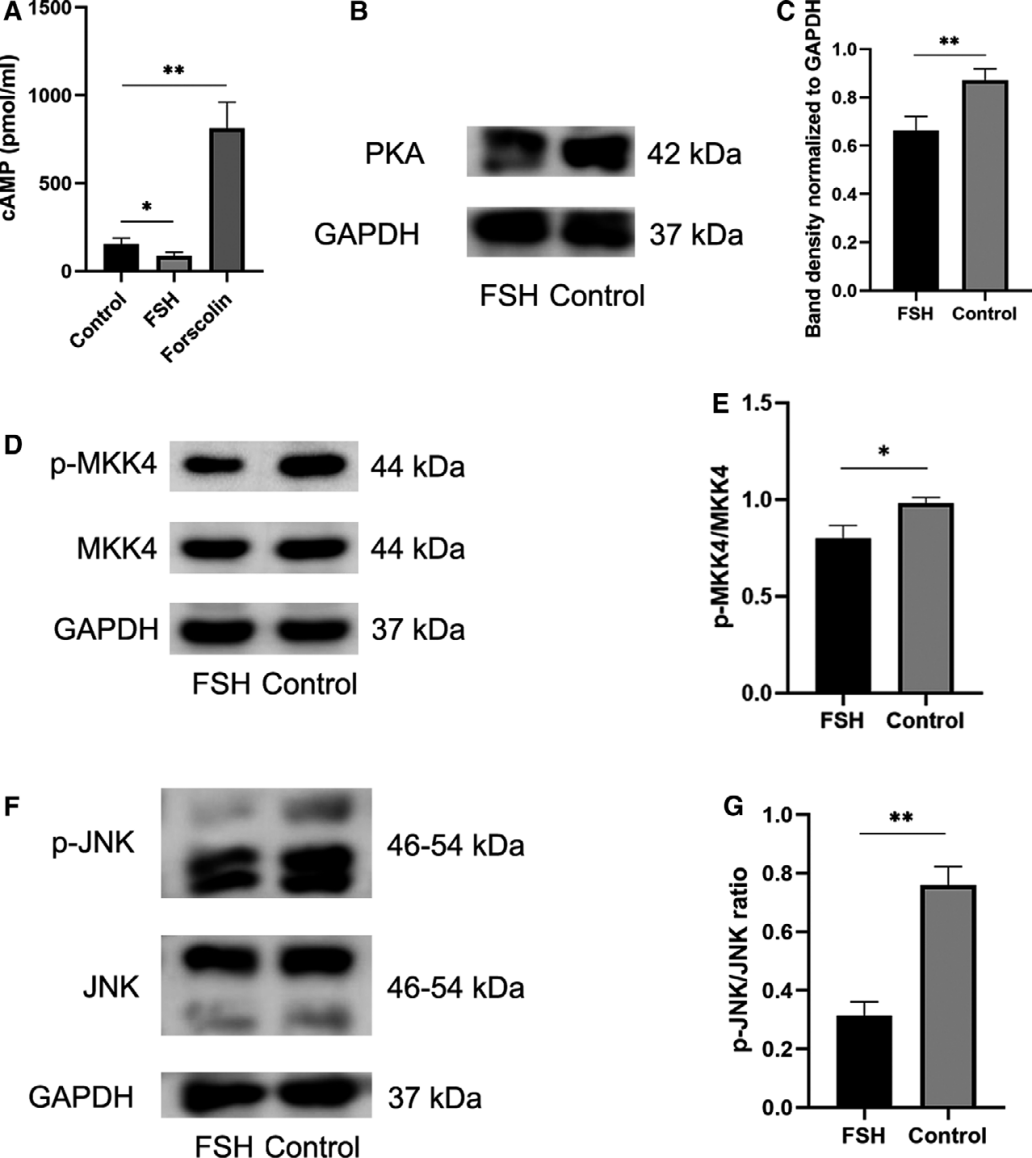
FSH inhibits cAMP/PKA and JNK pathways. (A) cAMP concentration of FSH group and control group measured by ELISA. (B, C) PKA expression and grayscale analysis of FSH group and control group measured by western blot. (D, E) MKK4 and p‐MKK4 expression and grayscale analysis of FSH group and control group measured by western blot. (F, G) JNK and p‐JNK expression and grayscale analysis of FSH group and control group measured by western blot. Statistical differences were determined by an unpaired Student’s *t*‐test (mean ± SEM, *n* = 3, *P < 0.05, **P < 0.01).

To further explore the pathways through which FSH affects chondrocytes, we validated the G protein‐coupled receptor downstream cAMP/PKA signaling pathway. It could be seen that after FSH stimulation, cAMP levels were reduced compared to the control group (refer to Fig. 4A), and western blot verified that PKA levels were similarly reduced in the FSH‐stimulated group (refer to Fig. 4B,C). In addition, we verified the changes in MKK4 and JNK (refer to Fig. 4D–G). Compared with the control groups, the total MKK4 and JNK levels were unchanged in the FSH‐stimulated groups, while the p‐MKK4 and p‐JNK levels appeared significantly decreased. The p‐MKK4/MKK4 ratio and p‐JNK/JNK ratio were significantly lower in the FSH groups than in the control groups, indicating that FSH can inhibit MKK4/JNK signaling pathway.

## Discussion

In previous studies, FSH has been shown to directly regulate bone mass, enhance osteoclast action and bone resorption and promote bone rebuilding [15]. The direct effect of FSH on cartilage has not been demonstrated, and the RNA‐Seq data analysis has provided us with several differential genes and related pathways.

After the RNA‐Seq analysis, the up‐regulated differential genes were mainly *Col12a1, Col1a1, Col5a1, Col3a1, Egr1*, etc. It was observed that the selected differential genes mainly encode collagen, which is an important component of ECM. Among the differential genes, it was observed that *EGR1* was directly associated with cartilage degeneration by comparing the normal and the OA‐afflicted joint cartilage, and the EGR1 expression was found to be elevated in the articular cartilage of OA patients [16, 17, 18]. *Col1a1* encodes the a1 chain of type I collagen, which is the most highly expressed type of collagen in the body, found mainly in bones, teeth, and other tissues. In normal cartilage tissue, the expression of type I collagen is rare. However, it has been reported that type I collagen is highly expressed in the synovium of OA patients [19], which suggests that *Col1a1* levels are closely related to the progression of OA. In the process of chondrocyte culture, it was found that normal round chondrocytes transform to polygonal shape, similar to fibroblasts, a phenomenon called dedifferentiation [20, 21]. And *Col1a1* is now widely acknowledged as a marker of chondrocyte dedifferentiation [22, 23, 24]. There is evidence that the increase in chondrocyte type I collagen enhances fibrotic remodeling in the upper middle layer of cartilage in patients with OA [25, 26]. Type I collagen disrupts the synthesis and stabilization of the ECM in OA [27]. Among the down‐regulated genes screened, *MGP* encodes matrix gla protein, a mineral‐binding protein synthesized by vascular smooth muscle and chondrocytes that inhibits cartilage calcification, and there is evidence that loss of matrix gla protein may have deleterious effects on chondrocytes, leading to elevated levels of some markers of catabolism, hypertrophy, and ossification [28]. In the study by Luo *et al*., [29] *MGP*‐deficient mice developed calcification of cartilage at the growth plate location and eventually fractures and other symptoms.

Both GO and KEGG are enriched in ECM‐related pathways, and cartilage, as a non‐vascular, non‐neural, non‐lymphatic connective tissue, can be divided into hyaline cartilage, fibrocartilage, and elastic cartilage [30]. All three types of cartilage secrete ECM [31]. Articular cartilage is composed of two main components: chondrocytes, which are responsible for the secretion and conversion of ECM components, and the ECM, which can confer biomechanical characteristics to articular cartilage, and the two work together to maintain homeostasis within the cartilage environment [32]. In OA, the balance between the synthesis and degradation of ECM components is altered, leading to pathological changes in the cartilage [33].

Synovial inflammation plays an important role as an essential pathological process, and the inflammatory factors can spread to the cartilage, causing upregulation of protease activity and leading to degradation of cartilage by matrix metalloproteinases and aggregation enzymes [34]. The degradation of multiple macromolecules in the ECM and the degradation of the ECM is closely related to the development of OA [35, 36, 37]. Previous studies have found that IL‐6, a cytokine that strongly activates the immune system and enhances the inflammatory response, is highly expressed in the animal models of spontaneous OA [38]. In the joints, IL‐6 is mainly secreted by the synovium, and in addition to causing a decrease in chondrocyte type II collagen, it is considered a key cytokine in the development of subchondral bone changes, promoting the formation of osteoclasts and bone resorption [38, 39]. Furthermore, IL‐6 can interact with IL‐1β and TNF‐α to further enhance the inflammatory effect [40, 41].

The FSHR is a G protein‐coupled receptor, and the cAMP/PKA signaling pathway, the most classical pathway, was first validated, and it was seen that FSH inhibited the cAMP/PKA pathway activity, and in a study with ethanol and 11β‐HSD2, researchers confirmed that the cAMP/PKA pathway regulates EGR1 expression [42]. What’s more, it has also been demonstrated that in some tissues, the cAMP/PKA pathway changes in the opposite direction to IL‐6, meaning that inhibition of the cAMP/PKA pathway activates the expression of IL‐6 [43, 44]. These evidences explained the specific mechanism of the elevated expression of EGR1 and IL‐6 in our experiment. In addition, we also validated JNK molecular activity in the MAPK family, as FSH can regulate the MAPK pathway in a non‐PKA‐dependent manner, whereas JNK has been shown to significantly regulate EGR1 [45]. MKK4, a JNK upstream protein belonging to the serine/threonine protein kinase family, has been shown to activate JNK by phosphorylating tyrosine residues [46]. Our experiments also showed that MKK phosphorylation levels were reduced after FSH stimulation, and the corresponding JNK molecule phosphorylation levels were also reduced, indicating that FSH can inhibit MKK4/JNK pathway activity in chondrocytes.

In conclusion, our data not only provide many differential genes for screening to investigate the effects of FSH on chondrocyte production, but also demonstrate that FSH can regulate EGR1, IL‐6, MGP and type I collagen through inhibition of the cAMP/PKA pathway, as well as MKK4/JNK pathway, causing chondrocyte dedifferentiation and cartilage inflammatory responses. This, therefore, explains the sudden increase in the prevalence of OA among menopausal women.

## Conflicts of interest

There is no conflict of interest.

## Author contributions

JX conceived and supervised the study; YW designed experiments; ZH and MZ performed experiments and analyzed data; ZH and YL wrote the manuscript; XZ and LK made manuscript revisions.

## Data Availability

The datasets analyzed during the current study are available from the corresponding author on reasonable request.
